# Supplementary material for: Endoplasmic reticulum-derived bodies enable a single-cell chemical defense in Brassicaceae plants
Source: Commun Biol. 2020 Jan 14;3:21. doi: 10.1038/s42003-019-0739-1 (PMC6959254; doi:10.1038/s42003-019-0739-1)
Supplement: Supplementary file 1 — Supplementary Information [file 42003_2019_739_MOESM1_ESM.pdf]

Supplementary Figures

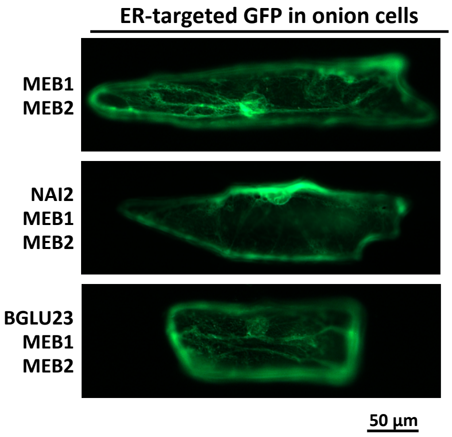

Supplementary Fig. 1 | MEB1 and MEB2 are unable to induce ER-body formation. Expression of the ER-body-membrane proteins MEB1 and MEB2 together with either NAI2 or BGLU23 do not induce the formation of ER bodies.

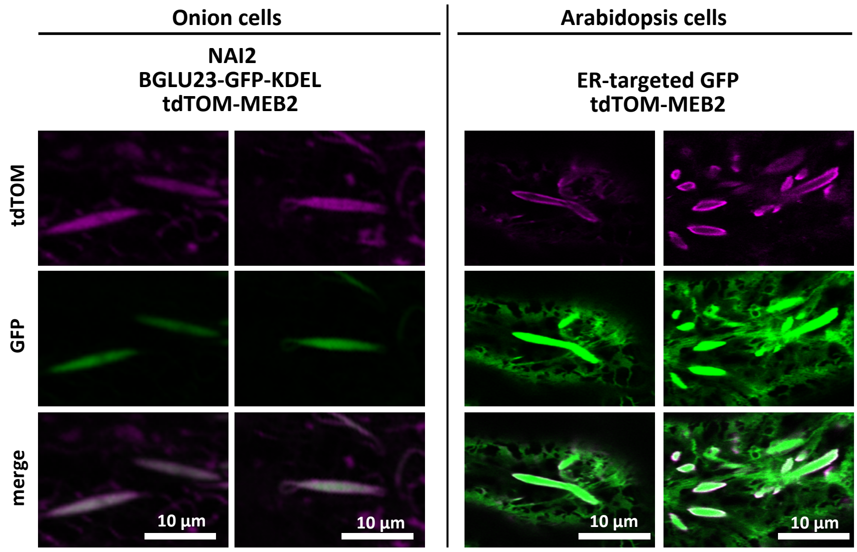

Supplementary Fig. 2 | The Brassicaceae-specific protein NAI2 induces the formation of ER-bodies that accumulate MEB2.

Additional two biological replicates with similar results to Fig. 2c. Fluorescence images of tdTomato-tagged ER-body-membrane protein MEB2 (tdTOM-MEB2), showing that the GFP-labeled ER-bodies are surrounded with the ER-body-membrane marker MEB2.

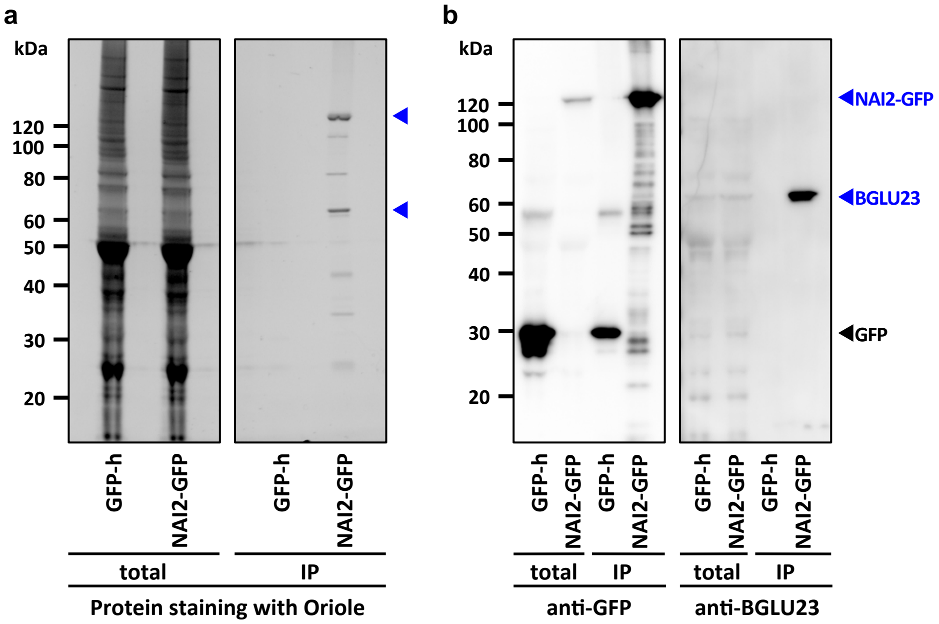

Supplementary Fig. 3 | Interaction between NAI2 and BGLU23.

a, Another biological replicate with similar results to Fig. 3b. Protein profiles of total homogenates (total) and immunoprecipitates with anti-GFP antibodies (IP) of 10-day-old seedlings of transgenic plants expressing GFP-HDEL (GFP-h) and transgenic plants expressing NAI2-GFP. Two major bands of immunoprecipitates of transgenic plants expressing NAI2-GFP are indicated by arrowheads.

b, Another biological replicate with similar results to Fig. 3c. Immunoblots of total homogenates (total) and immunoprecipitates with anti-GFP antibodies (IP) of 10-day-old seedlings of transgenic plants expressing GFP-HDEL (GFP-h) and transgenic plants expressing NAI2-GFP using either anti-GFP antibodies or anti-BGLU23 antibodies.

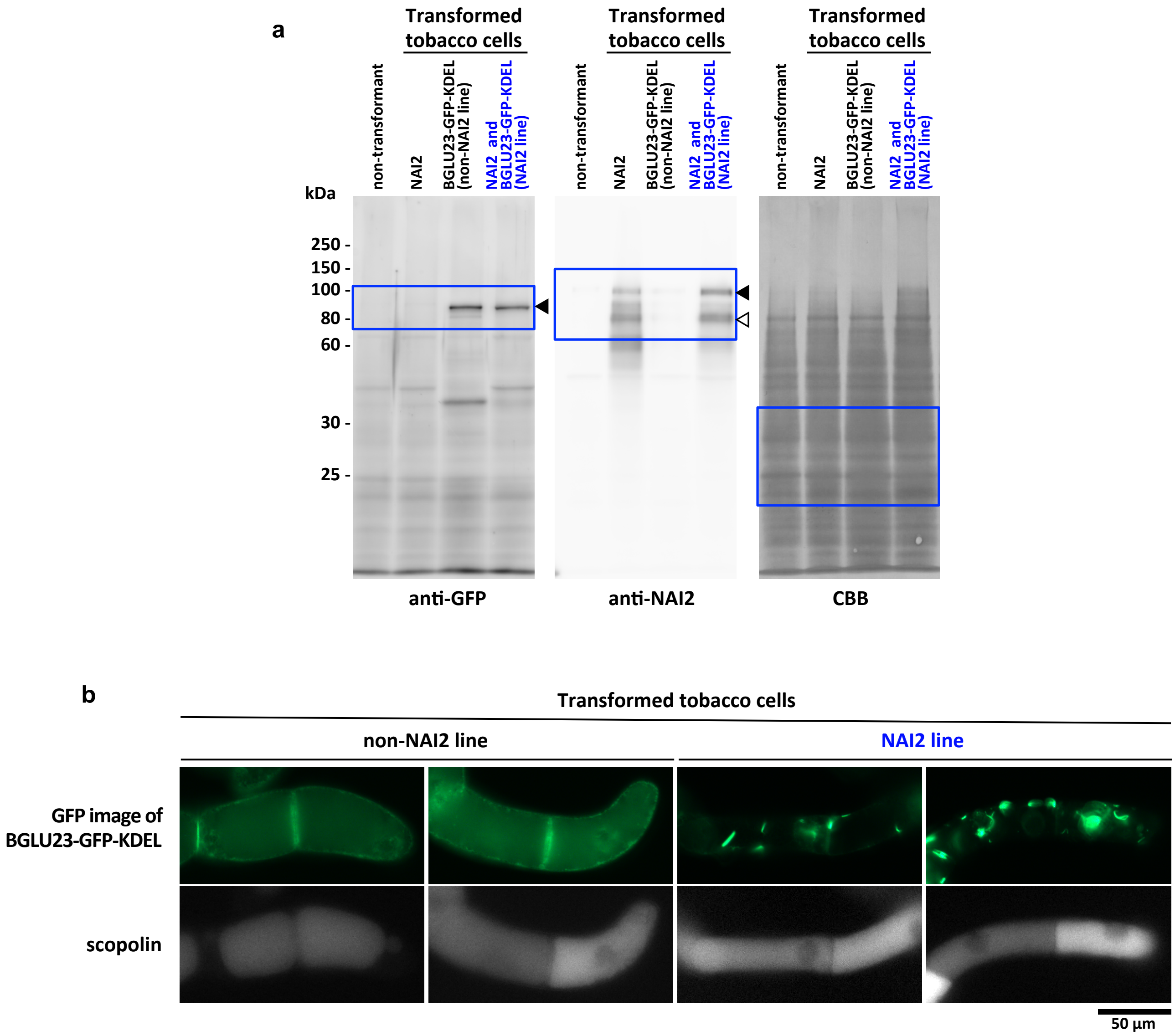

Supplementary Fig. 4 | ER-body formation prevents β-glucosidase from leaking into the vacuoles.

a, Full images of two immunoblots and a CBB-stained gel in Fig. 4a.

b, Additional two biological replicates with similar results to Fig. 4b. Fluorescence images of BGLU23-GFP-KDEL in NAI2 and non-NAI2 tobacco cell lines. The cells were exposed to scopoletin, leading to accumulation of scopolin, a BGLU23 substrate, in the vacuoles. Both scopoletin and scopolin are naturally fluorescent molecules. The non-NAI2 line accumulated BGLU23-GFP-KDEL in the vacuole, while the NAI2 line accumulated it in ER bodies. DIC, differential interference contrast images.

a

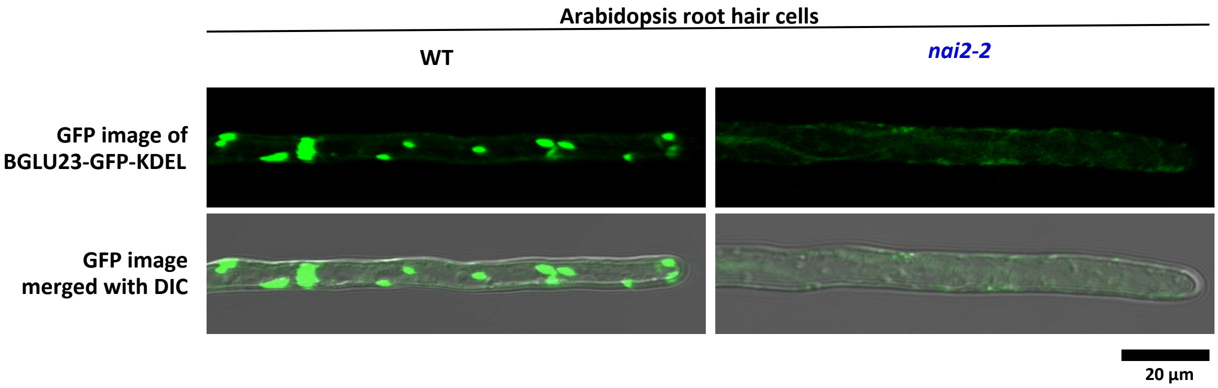

b

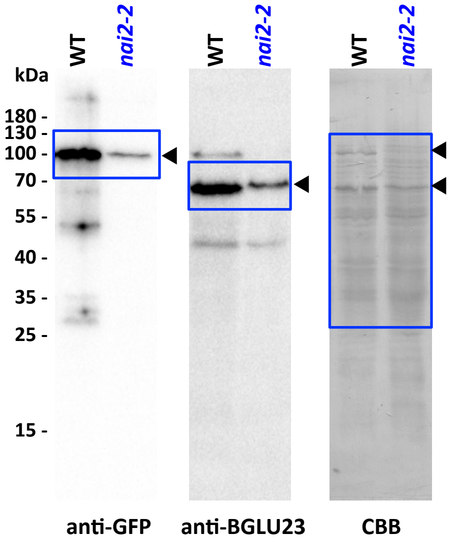

Supplementary Fig. 5 | NAI2 is required for accumulation of BGLU23 in ER bodies.

a, Another biological replicate with similar results to Fig. 5a. Fluorescence images of BGLU23-GFP-KDEL in *A. thaliana* root hair cells, showing that BGLU23-GFP-KDEL is accumulated in ER bodies of the wild type (WT), while it is accumulated in the vacuole of *nai2-2*. DIC, differential interference contrast images.

b, Full images of two immunoblots and a CBB-stained gel in Fig. 5b. Accumulation of BGLU23-GFP-KDEL and endogenous BGLU23 in 3-week-old wild type (WT) and *nai2-2*. Immunoblot with anti-GFP antibody shows BGLU23-GFP-KDEL protein level. Immunoblot with anti-BGLU antibody shows endogenous BGLU23 protein level. CBB staining as a loading control.

Supplementary Tables

Supplementary Table 1 | Abbreviations of glucosinolates identified.

Symbol	Name	Common name	Biosynthesis pathway from
4MTB	4-methylthiobutyl glucosinolate	Glucoerucin	Methionine
5MTP	5-methylthiopentyl glucosinolate	Glucoberteroin	Methionine
6MTH	6-methylthiohexyl glucosinolate	Glucolesquerellin	Methionine
7MTH	7-methylthioheptyl glucosinolate		Methionine
8MTO	8-methylthiooctyl glucosinolate		Methionine
4MSOB	4-methylsulfinylbutyl glucosinolate	Glucoraphanin	Methionine
5MSOP	5-methylsulfinylpentyl glucosinolate	Glucoalyssin	Methionine
7MSOH	7-methylsulfinylheptyl glucosinolate	Glucoibarin	Methionine
8MSOO	8-methylsulfinyloctyl glucosinolate	Glucohirsutin	Methionine
2PE	2-phenylethyl glucosinolate	Gluconasturtiin	Phenylalanine
3BzOP	3-benzoyloxypropyl glucosinolate	Glucomalcomiin	Phenylalanine, Methionine
4BzOB	4-benzoyloxybutyl glucosinolate		Phenylalanine, Methionine
5BzOP	5-benzoyloxypentyl glucosinolate		Phenylalanine, Methionine
I3M	Indol-3-ylmethyl glucosinolate	Glucobrassicin	Tryptophan
1MOI3M	1-methoxyindol-3-ylmethyl glucosinolate	Neoglucobrassicin	Tryptophan
4MOI3M	4-methoxyindol-3-ylmethyl glucosinolate	4-methoxyglucobrassicin	Tryptophan

Supplementary Table 2 | Primers used in this study.

Primer set name	Primer name	Sequence	Note
NAI2	At3g15950-cDNAFW	5'-CACCACAACACAGTGTATTTTGATC-3'	Amplification of *NAI2* cDNA
	At3g15950-RV	5'-TCAATTAAGTGAACTAAGAAACT-3'	
BGLU37/TGG2	TGG2-FW	5'-CACCATGCAACACAACACATACAT-3'	Amplification of *BGLU37* cDNA
	TGG2-RV	5'-TCATGTGAGGCTCTTCCTAT-3'	
CRT1b	CRT2-FW	5'-ATGGCGAAAATGATTCCTAGC-3'	Amplification of *CRT1b* cDNA
	CRT2-RV	5'-CTATAGCTCATCATGAGCGG-3'	
IAR3/JR3	IAR3/JR3-FW	5'-AATCCGAGATAAGTCATGAGTTT-3'	Amplification of *IAR3* cDNA
	IAR3/JR3-RV	5'-GTTGGCATCAAAGTTCATCTTTT-3'	
PYK10	PYK10-FW	5'-CCATGGTTTTGCAAAAGCTTCCTC-3'	Amplification of *BGLU23* cDNA
	PYK10-RV	5'-TTAAAGCTCATCCTTCTTGAGCGC-3'	
AtSH-EP	KDEL1-FW	5'-GACATGAAACTTTTCTTTATTGT-3'	Amplification of *AtSH-EP* cDNA
	KDEL1-RV	5'-CTAGAGCTCGTCTTTAACATC-3'	
AtHSP90.7/SHD	SHD-FW	5'-ATGAGGAAGAGGACGCTCGT-3'	Amplification of *AtHSP90.7* cDNA
	SHD-RV	5'-CTACAGTTCGTCCTTGGTGT-3'	
BIP2	Bip1/2-FW	5'-ATGGCTCGCTCGTTTGGAGC-3'	Amplification of *BIP2* cDNA
	Bip1/2-RV	5'-CTAGAGCTCATCGTGAGACT-3'	
PYK10-*Sal*I	PYK10-CFW	5'-GACAAGAAGGATGAGCTTTAA-3'	Creation of *Sal*I site of PYK10
	PYK10-CRV	5'-GACGAGCGCGGATGGACGA-3'	
GFP-*Sal*I	GFP-Sal/FW	5'-GTCGACATGGGCGGCATGGTGAGCAAGGGC-3'	Amplification of *GFP* cDNA at *Sal*I
	GFP-Sal/RV	5'-GTCGACATCGTGGTGGTGGTGGTGGTGCCC-3'	
NAI2g	attL1-NAI2_F1	5'-GGCTTTAAAGGAACCATGGGAACAAAGTTTTTAGCTC-3'	Amplification of NAI2 genomic sequence
	NAI2-attL2_R1	5'-CAAGAAAGCTGGGTCATTAAGTGAACTAAGAAACTCAACCC-3'	
pENTR1A	L1-Kzk_pENTR1A_R1	5'-GGTTCCTTTAAAGCCTGCTTTTTTG-3'	Amplification of pENTR1A backbone
	attL2_pENTR1A_F1	5'-GACCCAGCTTTCTTGTACAAAG-3'	
ProNAI2	attB4-ProNAI2_F1	5'-TGTATAGAAAAGTTGGTGTAAGTGTAAGTGTAACCAAAGGAG-3'	Amplification of NAI2 promoter with BP reaction adaptors
	ProNAI2-attB1r_R1	5'-TTTTGTACAAACTTGggtggaagattgttatcacaaagatc-3'	
BP adaptors	gggg-attB4_F1	5'-ggggacaactttgtatagaaaagttg-3'	Conjugation of the adaptors of BP reaction
	gggg-attB1r_R1	5'-ggggactgctttttgtacaaacttg-3'	
