# Supplementary material for: Endoplasmic reticulum-derived bodies enable a single-cell chemical defense in Brassicaceae plants
Source: Commun Biol. 2020 Jan 14;3:21. doi: 10.1038/s42003-019-0739-1 (PMC6959254; doi:10.1038/s42003-019-0739-1)
Supplement: Supplementary file 5 — Description of additional supplementary items [file 42003_2019_739_MOESM5_ESM.docx]

**Description of additional supplementary items**

**Supplementary Data 1 |** List of metabolites having a profile of I_WT,0_ > I_WT,30_ and I _bglu,30_ > I _WT,30_ by MS signal.

**Supplementary Data 2** | Mass-spectrometry data for proteomes identified by co-immunoprecipitation of GFP-HDEL (GFP-h) or NAI2-GFP.

**Supplementary Data 3** | Source data used for dot-plot format in this study.
