# Supplementary material for: Endoplasmic reticulum-derived bodies enable a single-cell chemical defense in Brassicaceae plants
Source: Commun Biol. 2020 Jan 14;3:21. doi: 10.1038/s42003-019-0739-1 (PMC6959254; doi:10.1038/s42003-019-0739-1)
Supplement: Supplementary file 6 — Reporting Summary [file 42003_2019_739_MOESM6_ESM.pdf]

nature research

Corresponding author(s): Ikuko Hara-Nishimura, Mikio Nishimura, Kenji Yamada

Last updated by author(s): Dec 3, 2019

Reporting Summary

Nature Research wishes to improve the reproducibility of the work that we publish. This form provides structure for consistency and transparency in reporting. For further information on Nature Research policies, see Authors & Referees and the Editorial Policy Checklist.

Statistics

For all statistical analyses, confirm that the following items are present in the figure legend, table legend, main text, or Methods section.

n/a	Confirmed	
☐	☒	The exact sample size (n) for each experimental group/condition, given as a discrete number and unit of measurement
☐	☒	A statement on whether measurements were taken from distinct samples or whether the same sample was measured repeatedly
☐	☒	The statistical test(s) used AND whether they are one- or two-sided<br>*Only common tests should be described solely by name; describe more complex techniques in the Methods section.*
☐	☒	A description of all covariates tested
☐	☒	A description of any assumptions or corrections, such as tests of normality and adjustment for multiple comparisons
☐	☒	A full description of the statistical parameters including central tendency (e.g. means) or other basic estimates (e.g. regression coefficient) AND variation (e.g. standard deviation) or associated estimates of uncertainty (e.g. confidence intervals)
☐	☒	For null hypothesis testing, the test statistic (e.g. F, t, r) with confidence intervals, effect sizes, degrees of freedom and P value noted<br>*Give P values as exact values whenever suitable.*
☒	☐	For Bayesian analysis, information on the choice of priors and Markov chain Monte Carlo settings
☒	☐	For hierarchical and complex designs, identification of the appropriate level for tests and full reporting of outcomes
☒	☐	Estimates of effect sizes (e.g. Cohen's d, Pearson's r), indicating how they were calculated

Our web collection on statistics for biologists contains articles on many of the points above.

Software and code

Policy information about availability of computer code

Data collection	n/a
Data analysis	Microsoft excel for statistical tests; ZEN of Carl ZEISS for microscopic analysis; ImageJ for area calculation in graphic images; R software for metbolomic analysis; SEQUEST (Thermo Fisher Scientific) for proteomic analysis.

For manuscripts utilizing custom algorithms or software that are central to the research but not yet described in published literature, software must be made available to editors/reviewers. We strongly encourage code deposition in a community repository (e.g. GitHub). See the Nature Research guidelines for submitting code & software for further information.

Data

Policy information about availability of data

All manuscripts must include a data availability statement. This statement should provide the following information, where applicable:

- Accession codes, unique identifiers, or web links for publicly available datasets
- A list of figures that have associated raw data
- A description of any restrictions on data availability

Nucleotide and protein sequence data are available form the GenBank/EMBL and UniProt database with following IDs: NAI1/At2g22770 (Gene ID, 816807; UniProt entry, Q8S3F1), NAI2/At3g15950 (820839; Q9LSB4), PYK10/BGLU23/At3g09260 (820082; Q9SR37), BGLU21/At1g66270 (842944; Q9C525), MEB1/At4g27860 (828899; Q8W4P8), and MEB2/At5g24290 (832496; F4KFS7). Proteome data were deposited in PRIDE (PXD016606). Metabolome data were deposited in MetaboLights with accession number (MTBLS1383). All other data supporting the findings of this study and newly generated plasmids in this study are available from the corresponding author upon reasonable request.

Field-specific reporting

Please select the one below that is the best fit for your research. If you are not sure, read the appropriate sections before making your selection.

☒ Life sciences ☐ Behavioural & social sciences ☐ Ecological, evolutionary & environmental sciences

For a reference copy of the document with all sections, see nature.com/documents/nr-reporting-summary-flat.pdf

Life sciences study design

All studies must disclose on these points even when the disclosure is negative.

Sample size	Sample sizes in our experiments were determined by the general standard used for plant science.
Data exclusions	No data was excluded from the analysis.
Replication	Each experiment in our paper was repeated in at least three times. Reproducibility is described in Methods.
Randomization	Each experiment in our paper was repeated in at least three times. Reproducibility is described in Methods.
Blinding	Investigators were not blinded.

Reporting for specific materials, systems and methods

We require information from authors about some types of materials, experimental systems and methods used in many studies. Here, indicate whether each material, system or method listed is relevant to your study. If you are not sure if a list item applies to your research, read the appropriate section before selecting a response.

Materials & experimental systems

n/a	Involved in the study	
☐	☒	Antibodies
☐	☒	Eukaryotic cell lines
☒	☐	Palaeontology
☐	☒	Animals and other organisms
☒	☐	Human research participants
☒	☐	Clinical data

Methods

n/a	Involved in the study	
☒	☐	ChIP-seq
☒	☐	Flow cytometry
☒	☐	MRI-based neuroimaging

Antibodies

Antibodies used	Antibodies against GFP (JL8; Clontech, CA, USA) and against BGLU23 (PYK10-IM) were used. Anti-BGLU23 (PYK10) antibodies that we generated were published in our previous paper (Plant J, 33: 493, 2003; PMID: 12581307) and then deposited at Bio Academia (Japan; https://www.bioacademia.co.jp/index.php).
Validation	The information about the specificity and titer of anti-BGLU23 antibody is available at the web site at Bio Academia (Japan; https://www.bioacademia.co.jp/product_list.php?srch_keyword=PYK10) and in our paper (Plant J, 33: 493, 2003; PMID: 12581307).

Eukaryotic cell lines

Policy information about cell lines

Cell line source(s)	tobacco cultured cells (BY-2).
Authentication	BY-2 cells are the suspension culture cells that are most widely used in plant science as a model plant system. The cell line was donated by authorized resource in Nagoya University.
Mycoplasma contamination	Impossible, because BY-2 cells are not derived from animals.
Commonly misidentified lines (See ICLAC register)	None.

Animals and other organisms

Policy information about studies involving animals; ARRIVE guidelines recommended for reporting animal research

Laboratory animals	n/a
Wild animals	Woodlice (Armadillidium vulgare).
Field-collected samples	Woodlice (Armadillidium vulgare) were collected in the Botanical Garden of Kyoto University and the backyard of Małpolska Centre of Biotechnology, Jagiellonian University. See Methods for the relevant details.
Ethics oversight	No ethical approval or guidance was required because woodlice are not harmful and are popular as terrarium pets. They are widely studies in evolutionary biology, behavioural ecology and so on.

Note that full information on the approval of the study protocol must also be provided in the manuscript.
